# Use of deep artificial neural networks to identify stroke during triage via subtle changes in circulating cell counts

**DOI:** 10.1186/s12883-022-02726-x

**Published:** 2022-06-03

**Authors:** Grant C. O’Connell, Kyle B. Walsh, Christine G. Smothers, Suebsarn Ruksakulpiwat, Bethany L. Armentrout, Chris Winkelman, Truman J. Milling, Steven J. Warach, Taura L. Barr

**Affiliations:** 1 School of Nursing, Case Western Reserve University, 10900 Euclid Avenue, Cleveland, OH 44106-4904 USA; 2 Molecular Biomarker Core, Case Western Reserve University, Cleveland, OH USA; 3 Department of Emergency Medicine, College of Medicine, University of Cincinnati, Cincinnati, OH USA; 4 Gardner Neuroscience Institute, University of Cincinnati, Cincinnati, OH USA; 5 Dell Seton Medical Center, University of Texas, Austin, TX USA; 6 School of Nursing, Ohio State University, Columbus, OH USA; 7 Valtari Bio Incorporated, Morgantown, WV USA

**Keywords:** Stroke, Immune system, NLR, Digital health, WBC, Machine-learning, Emergency medicine, Stroke scales, Decision support, Triage

## Abstract

**Background:**

The development of tools that could help emergency department clinicians recognize stroke during triage could reduce treatment delays and improve patient outcomes. Growing evidence suggests that stroke is associated with several changes in circulating cell counts. The aim of this study was to determine whether machine-learning can be used to identify stroke in the emergency department using data available from a routine complete blood count with differential.

**Methods:**

Red blood cell, platelet, neutrophil, lymphocyte, monocyte, eosinophil, and basophil counts were assessed in admission blood samples collected from 160 stroke patients and 116 stroke mimics recruited from three geographically distinct clinical sites, and an ensemble artificial neural network model was developed and tested for its ability to discriminate between groups.

**Results:**

Several modest but statistically significant differences were observed in cell counts between stroke patients and stroke mimics. The counts of no single cell population alone were adequate to discriminate between groups with high levels of accuracy; however, combined classification using the neural network model resulted in a dramatic and statistically significant improvement in diagnostic performance according to receiver-operating characteristic analysis. Furthermore, the neural network model displayed superior performance as a triage decision making tool compared to symptom-based tools such as the Cincinnati Prehospital Stroke Scale (CPSS) and the National Institutes of Health Stroke Scale (NIHSS) when assessed using decision curve analysis.

**Conclusions:**

Our results suggest that algorithmic analysis of commonly collected hematology data using machine-learning could potentially be used to help emergency department clinicians make better-informed triage decisions in situations where advanced imaging techniques or neurological expertise are not immediately available, or even to electronically flag patients in which stroke should be considered as a diagnosis as part of an automated stroke alert system.

## Introduction

Quick and confident recognition of stroke in the emergency department dramatically increases the odds of intervention and positive outcome by facilitating timely intra or inter-hospital referral to stroke-specific care [1]. Unfortunately, the tools available to emergency department clinicians for stroke recognition can be limited. In smaller rural hospitals in particular, there is often not on-demand access to the advanced neuroradiological imaging techniques and expertise needed to reliably detect early stroke pathology, especially in the case of ischemic events [2, 3]. In such situations, critical early triage decisions are made using symptom-based stroke recognition and severity scales, which can be limited in their accuracy [4, 5]. Furthermore, even in larger hospitals where advanced imaging techniques are more readily available, patients can be delayed in receiving such imaging due to a failure of the emergency medicine team to initially consider stroke as a diagnosis, especially in cases presenting with mild, ambiguous, or non-traditional symptoms [6]. As a result, anywhere from 10 to 25% of stroke patients are misdiagnosed at initial assessment in the emergency department, leading to life-threatening delays in care [7, 8]. Thus, if tools can be developed which can identify stroke using widely available and commonly used laboratory measures, they could be employed to help emergency department clinicians make better informed triage decisions when stroke is suspected, or even to automatically flag patients whom stroke should be considered as a possible diagnosis.

The complete blood count with differential (CBC + diff) is one of the most commonly ordered laboratory tests requested in the emergency department [9]; it can be performed in virtually any hospital lab, and increasingly at the point of care [10]. Stroke is pathophysiologically associated with a multitude of changes to the cellular composition of the peripheral blood. For example, stroke triggers robust activation of the innate arm of the immune system, and simultaneous suppression of the adaptive arm of the immune system [11, 12]; this phenomenon has been shown to result in a rise in the circulating counts of innate immune cells such as neutrophils and monocytes, and a decrease in the circulating counts of adaptive immune cells such as lymphocytes [13]. Furthermore, several studies have reported altered counts of red blood cells and platelets in stroke [14, 15], which is not surprising given their role in coagulation and thrombosis. Therefore, it is possible that stroke could be identified in the emergency department using stroke associated patterns of changes to the CBC + diff.

A recent study by Onder et al. [16] reported that the ratio between circulating neutrophil count and lymphocyte count can be used to identify stroke at hospital admission, but with limited accuracy. However, the neutrophil-to-lymphocyte ratio (NLR) is a simplistic metric, and fails to account for changes in counts of other cell populations measured as part of the CBC + diff, and the potential advantage of considering them all collectively. Thus, analysis considering the entirety of the CBC + diff using advanced pattern recognition techniques may be able to offer significantly greater levels of diagnostic performance. Artificial neural networks are biologically-inspired machine-learning algorithms that can be used to solve multivariate classification problems involving complex nonlinear or indirect relationships [17]. In this study, our aim was to determine whether a machine-learning strategy employing artificial neural networks can be implemented to identify stroke during triage using stroke-induced patterns of changes to circulating blood cell counts routinely measured as part of the CBC + diff.

## Methods

### Experimental design

A cohort of 160 patients with stroke and 116 stroke mimics were recruited from three geographically distinct tertiary care hospitals (Ruby Memorial Hospital, Morgantown, WV; University of Cincinnati Medical Center, Cincinnati, OH; Dell-Seton Medical Center, Austin, TX) between 2011 and 2019. Counts of red blood cells, platelets, neutrophils, lymphocytes, monocytes, eosinophils, and basophils were determined in peripheral blood samples collected at emergency department admission. An ensemble neural network model discriminating between stroke patients and stroke mimics using the collective counts of all seven aforementioned cell populations as input was developed in a randomly selected training set comprised of 75% of the total subject pool, and validated in a test set comprised of the remaining 25% of the total subject pool. The diagnostic performance of this ensemble neural network model was then compared to the diagnostic performance of the counts of each individual cell population, as well as NLR, using receiver operating characteristic (ROC) analysis. Finally, the potential clinical benefit of using the ensemble neural network model as a triage decision making tool as opposed to symptom-based assessments such as the Cincinnati Prehospital Stroke Scale (CPSS) and the National Institutes of Health Stroke Scale (NIHSS) was evaluated via decision curve analysis.

### Patients

All patients admitted as suspected strokes were considered for enrolment, pending that study staff were available for blood collection. Patients who displayed definitive radiographic evidence of ischemic or hemorrhagic pathology on magnetic resonance imaging (MRI) or computed tomography (CT) were identified as strokes. Patients receiving a final definitive negative diagnosis for stroke upon neuroradiological imaging and clinical evaluation were identified as acute stroke mimics [18]. All diagnoses were adjudicated by an experienced stroke physician. Patients were excluded if they received a non-definitive diagnosis, were diagnosed with transient ischemic attack, reported a prior hospitalization within 30 days, were under 18 years of age, or were more than 24 h past symptom onset. Time from symptom onset was determined by the time the patient was last known to be free of neurological symptoms. Demographic information was collected from either the subject or legally authorized representative by a trained clinician.

### Stroke scale scores

NIHSS scores were collected by a member of the clinical care team at the time of blood draw. CPSS scores were retrospectively calculated from individual NIHSS items similar to the methodology employed by Purrucker et al. [5] and Tarkanyi et. al. [19]. Specifically, values of greater than zero on item 4 of the NIHSS (facial palsy) were scored as abnormal for the facial droop item of the CPSS. Values of greater than zero on item 5 of the NIHSS (R/L arm motor drift) were scored as abnormal for the arm drift item of the CPSS. Finally, values of greater than 0 on either items 9 or 10 of the NIHSS (language and dysarthria, respectively) were scored as abnormal for the speech item of the CPSS.

### Circulating cell counts

Venous blood was collected in K_2_EDTA vacutainers (Becton Dickenson, Franklin Lakes, NJ) and immediately submitted to the clinical pathology laboratory for CBC + diff testing as described previously [20]. Results were subsequently retrieved from the medical record. NLR was calculated as absolute neutrophil count divided by absolute lymphocyte count [16]. In situations where the counts of a cell population were too low for detection, zero values were used for downstream analyses.

### Artificial neural network

Neural network analysis was performed in R version 3.6 [21] using the ‘neuralnet’ and ‘NeuralNetTools’ packages [22]. An ensemble neural network model was developed discriminating between stroke patients and stroke mimics in the training set using the counts of all seven cell populations as input. This ensemble model was comprised of five individual feedforward neural network sub-models, each constructed using identical architecture, which was selected based on a simple preliminary grid search of hyper-parameters. Each sub-model of the ensemble used the sigmoid activation function, and consisted of an input layer with 7 nodes accepting 0–1 scaled absolute cell counts, two hidden layers with 5 and 3 nodes respectively, and an output layer with a single node producing a predicted probability of stroke. These sub-models used were trained via backpropagation using random starting weights and an error threshold of 0.25 cross entropy to determine convergence. For each subject, the predicted probabilities of stroke generated across the sub-models were pooled by simple averaging to yield an ensemble predicted probability of stroke. The ensemble predicted probabilities of stroke were then subjected to ROC analysis to determine diagnostic performance. The diagnostic performance of the final ensemble model was subsequently evaluated in the test set, and statistically compared to that observed in the training set.

To gain insights into the associations between the counts of each cell population and stroke prediction probability in the final ensemble neural network model, Olden relative importance values [23] were generated for each sub-model and averaged.

### Statistics

All statistics were performed in R version 3.6. Mann–Whitney U test was used to compare continuous variables, while Fisher’s exact test was used to compare categorical variables. Stratified U testing was performed using the ‘coin’ package [24]. The strength and significance of correlative relationships were assessed using Spearman’s rho.

The performance of continuous variables in binary classification was assessed via ROC analysis using the ‘pROC’ package [25]. Sensitivity and specificity values associated with the diagnostic cutoff yielding the highest combined value (Youden index) were reported. The bootstrapping method described by DeLong et al. was used to calculate 95% confidence intervals for area under curve (AUC) values, test the null hypothesis that AUC values = 0.5, compare AUC values from different ROC curves, and generate 95% confidence intervals for sensitivity and specificity values [26]. 1,000 bootstrap samples were used for all bootstrap calculations.

Decision curve analysis [27] was performed using the ‘dca’ package (https://github.com/ddsjoberg/dca). The predicted probabilities of stroke generated by the neural network ensemble were used to generate decision curves directly, while CPSS and NIHSS scores were first converted to prediction probabilities via binary logistic regression, which were subsequently used for decision curve generation. Decision curves were generated across threshold probabilities ranging from 0–1 and were smoothed via moving regression using a span value of 0.25.

In the case of all statistical testing, the null hypothesis was rejected when *p* < 0.05. The sample size was arbitrarily determined based on availability of resources. The parameters of all statistical tests performed are outlined in detail within the figure legends.

## Results

### Clinical and demographic characteristics

Across the total study sample, 84% of stroke cases were ischemic, and the remaining 16% were hemorrhagic. Stroke patients were significantly older than stroke mimics, but relatively well matched in terms of sex, race, and the prevalence of cardiovascular disease risk factors such as hypertension, diabetes, and dyslipidemia. Unsurprisingly, stroke patients displayed significantly higher scores on both the CPSS and the NIHSS than stroke mimics. Similar clinical demographic trends were observed within the subsets of patients comprising both the training and test sets used for neural network model development and evaluation (Table 1). The stroke mimic group displayed a relatively wide variety of final diagnoses, most common of which were seizure, complex migraine, hypertensive encephalopathy, psychogenic or psychiatric pathologies including conversion disorder, hypotension, and infection (Table 2).

**Table 1:** Clinical and demographic characteristics

	**All subjects (*n* = 276)**			**Training set (*n* = 207)**			**Test set (*n* = 69)**		
	**Mimic** **(*n* = 116)**	**Stroke (*n* = 160)**	***p*-value**	**Mimic** **(*n* = 87)**	**Stroke (*n* = 120)**	***p*-value**	**Mimic** **(*n* = 29)**	**Stroke** **(*n* = 40)**	***p*-value**
^**a**^**Age *median (IQR)***	58 (49–70)	71 (60–81)	< 0.001*	58 (48–68)	71 (60–82)	< 0.001*	66 (54–73)	69 (59–80)	0.184
^**b**^**Female *n (%)***	65 (56.0)	73 (45.6)	0.113	47 (54.0)	57 (47.5)	0.399	18 (62.1)	16 (40.0)	0.090
^**b**^**American Indian *n (%)***	2 (1.7)	0 (0.0)	0.176	1 (1.1)	0 (0.0)	0.420	1 (3.4)	0 (0.0)	0.420
^**b**^**White *n (%)***	93 (80.2)	137 (85.6)	0.254	72 (82.8)	99 (82.5)	1.000	21 (72.4)	38 (95.0)	0.014*
^**b**^**Asian *n (%)***	0 (0.0)	1 (0.6)	1.000	0 (0.0)	1 (0.8)	1.000	0 (0.0)	0 (0.0)	1.000
^**b**^**Black / African American *n (%)***	21 (18.1)	22 (13.8)	0.401	14 (16.1)	20 (16.7)	1.000	7 (24.1)	2 (5.0)	0.030*
**Ischemic stroke *n (%)***	-	135 (84.4)	-	-	103 (85.8)	-	-	32 (80.0)	-
**Hemorrhagic stroke *n (%)***	-	25 (15.6)	-	-	17 (14.2)	-	-	8 (20.0)	-
^**a**^**CPSS *median (IQR)***	1 (0–1)	1.5 (0–3)	< 0.001*	1 (0–1)	2 (1–3)	< 0.001*	1 (0–1)	1 (0–3)	0.390
^**a**^**NIHSS *median (IQR)***	2 (0–5)	7 (2–14)	< 0.001*	2 (1–5)	7 (2–13)	< 0.001*	3 (2–5)	7.5 (1–15)	0.038*
^**a**^**Time from onset *median (IQR)***	241 (145–467)	282 (135–651)	0.222	236 (137–458)	282 (142–651)	0.089	264 (195–498)	306 (110–647)	0.617
^**b**^**Hypertension *n (%)***	72 (62.1)	115 (71.9)	0.091	51 (58.6)	90 (75.0)	0.016*	21 (72.4)	25 (62.5)	0.446
^**b**^**Dyslipidemia *n (%)***	48 (41.4)	71 (44.4)	0.625	33 (37.9)	50 (41.7)	0.667	15 (51.7)	21 (52.5)	1.000
^**b**^**Diabetes *n (%)***	35 (30.2)	41 (25.6)	0.416	28 (32.2)	33 (27.5)	0.537	7 (24.1)	8 (20.0)	0.771
^**b**^**Previous stroke *n (%)***	23 (19.8)	38 (23.8)	0.466	19 (21.8)	32 (26.7)	0.514	4 (13.8)	6 (15)	1.000
^**b**^**Previous TIA *n (%)***	16 (13.8)	17 (10.6)	0.456	9 (10.3)	14 (11.7)	0.826	7 (24.1)	3 (7.5)	0.082
^**b**^**History of atrial fibrillation *n (%)***	11 (9.5)	34 (21.3)	0.013*	7 (8.0)	23 (19.2)	0.028*	4 (13.8)	11 (27.5)	0.240
^**b**^**History of myocardial infarction *n (%)***	12 (10.3)	26 (16.3)	0.215	8 (9.2)	21 (17.5)	0.106	4 (13.8)	5 (12.5)	1.000
^**b**^**Hypertension medication *n (%)***	66 (56.9)	100 (62.5)		45 (51.7)	78 (65.0)	0.063	21 (72.4)	22 (55.0)	0.208
^**b**^**Cholesterol medication *n (%)***	41 (35.3)	67 (41.9)		30 (34.5)	50 (41.7)	0.315	11 (37.9)	17 (42.5)	0.806
^**a**^**Anticoagulant or antiplatelet *n (%)***	48 (41.4)	82 (51.3)		35 (40.2)	63 (52.5)	0.092	13 (44.8)	19 (47.5)	1.000
^**b**^**Smoking *n (%)***	24 (20.7)	40 (25.0)	0.471	16 (18.4)	33 (27.5)	0.139	8 (27.6)	7 (17.5)	0.382

^a^Intergroup comparison of medians via Mann–Whitney U-Test; ^b^Intergroup comparison of proportions via Fisher’s exact test; *Statistically significant

**Table 2:** Final diagnoses of stroke mimics

	**Total sample (*n* = 116)**	**Training set (*n* = 87)**	**Test set (*n* = 29)**
Seizure *n (%)*	20 (17.2)	16 (18.4)	4 (13.8)
Complex migraine *n (%)*	13 (11.2)	8 (9.2)	5 (17.2)
Hypertensive encephalopathy *n (%)*	7 (6.0)	5 (5.7)	2 (6.9)
Psychogenic event / psychiatric disorder *n (%)*	7 (6.0)	6 (6.9)	1 (3.4)
Hypotension / syncope *n (%)*	7 (6.0)	5 (5.7)	2 (6.9)
Sepsis / infection *n (%)*	7 (6.0)	6 (6.9)	1 (3.4)
Adverse drug reaction *n (%)*	6 (5.2)	4 (4.6)	2 (6.9)
Space occupying lesion *n (%)*	6 (5.2)	4 (4.6)	2 (6.9)
Bell's palsy *n (%)*	5 (4.3)	4 (4.6)	1 (3.4)
Alcohol intoxication *n (%)*	4 (3.4)	2 (2.3)	2 (6.9)
Hypoglycemia *n (%)*	3 (2.6)	2 (2.3)	1 (3.4)
Dementia *n (%)*	3 (2.6)	3 (3.4)	0 (0.0)
Dehydration *n (%)*	3 (2.6)	3 (3.4)	0 (0.0)
Vestibular dysfunction *n (%)*	3 (2.6)	1 (1.1)	2 (6.9)
Peripheral neuropathy *n (%)*	2 (1.7)	2 (2.3)	0 (0.0)
Transient global amnesia *n (%)*	2 (1.7)	1 (1.1)	1 (3.4)
Parkinson’s disease *n (%)*	2 (1.7)	2 (2.3)	0 (0.0)
Multiple sclerosis *n (%)*	2 (1.7)	2 (2.3)	0 (0.0)
Hepatic encephalopathy *n (%)*	2 (1.7)	1 (1.1)	1 (3.4)
Other *n (%)*	12 (10.3)	10 (11.5)	2 (6.9)

### Differences in circulating cell counts

**Fig. 1:**
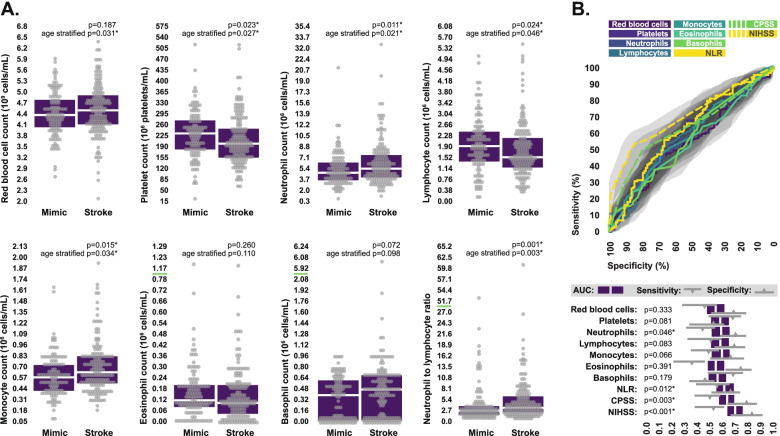
Comparison of circulating cell counts between stroke patients and stroke mimics. **A** Circulating red blood cell, platelet, neutrophil, lymphocyte, monocyte, eosinophil, and basophil counts, as well as neutrophil-to-lymphocyte ratios in stroke patients and stroke mimics at hospital admission. Bloxplots indicate median and interquartile range. Some axes are broken to display extreme values, as indicated by dashed line. Intergroup statistical comparisons were performed via two-sided Mann–Whitney U test, both stratified and unstratified by age quartile. **B** ROC curves depicting the individual abilities of circulating cell counts to discriminate between stroke patients and stroke mimics, compared to the CPSS and the NIHSS. Sensitivity and specificity values are indicated for the diagnostic cutoff which produced the highest Youden index. Bootstrapped 95% confidence intervals associated with all diagnostic statistics are indicated. *P*-values indicate the probability that area under ROC curve values differ from 0.5. *Statistically significant

Across the total study sample, neutrophil, monocyte, and red blood cell counts were significantly elevated in stroke patients compared to stroke mimics, independent of age. Basophil counts were also higher in the stroke group, but this difference was not statistically significant. Platelet and lymphocyte counts were both significantly lower in stroke patients compared to stroke mimics. Eosinophil counts were also lower in the stroke group, however this difference was not statistically significant. Unsurprisingly given the differences we observed in both neutrophil and lymphocyte counts, NLR was significantly higher in stroke patients relative to stroke mimics (Fig. 1A).

Despite many of these differences being statistically significant, none were adequate to discriminate between groups with high levels of accuracy when considered in isolation. The counts of the individual cell populations performed poorly in ROC analysis, producing AUC values ranging from 0.54–0.59, sensitivities ranging from 24.4–68.1%, and specificities ranging from 45.7–85.3%. NLR exhibited slightly better diagnostic performance than the counts of any individual cell population, producing an AUC of 0.61 (0.95 CI: 0.55–0.68), but could still only discriminate between groups with 55.6% (0.95 CI: 39.3–65.0%) sensitivity and 66.4% (0.95 CI: 50.8–74.1%) specificity. Comparatively, CPSS scores produced an AUC of 0.63 (0.95 CI: 0.57–0.670), and could differentiate between groups with 50.0% (0.95 CI: 36.8–58.1%) sensitivity and 68.0% (0.95 CI: 50.9–76.7%) specificity, while NIHSS scores produced an AUC of 0.70 (0.95 CI: 0.64–0.76) and could differentiate between groups with 53.1% (0.95 CI: 38.1–60.9%) sensitivity and 84.5% (0.95 CI: 65.8–91.2%) specificity (Fig. 1B).

### Artificial neural network performance

**Fig. 2:**
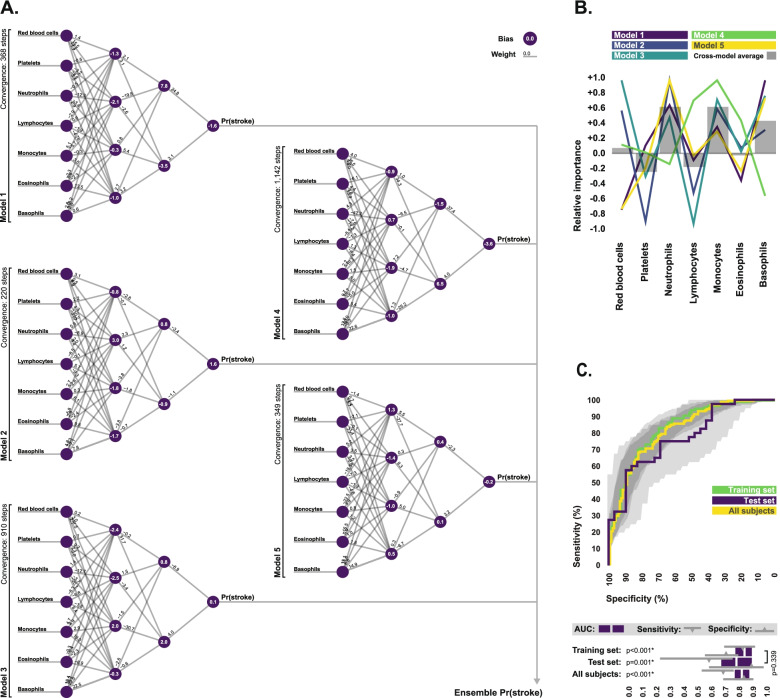
Final ensemble neural network model. **A** Visual representation of the final ensemble neural network model developed in the training set, which was comprised of five individual sub-models. The synaptic weights and node bias terms associated with each of the five sub-models are indicated. All input values were scaled between 0 and 1 for training. Sigmoid activation functions were used at all nodes. Final ensemble stroke prediction probabilities were generated via simple averaging of the sub-model prediction probabilities. Pr, probability. **B** The relative importance of each cell type in each of the five individual sub-models which comprise the final ensemble neural network model, as calculated using the Olden method. Averaged importance values for each cell type across the five sub-models are also indicated. Values of 0 indicate little importance, while values approaching 1 and -1 indicate strong positive and negative associations with stroke prediction probability respectively. **C** ROC curves depicting the ability of the final ensemble neural network model to discriminate between stroke patients and controls in the training set, test set, and total study population. Sensitivity and specificity values are indicated for the diagnostic cutoff which produced the highest Youden index. Bootstrapped 95% confidence intervals associated with all diagnostic statistics are indicated. *P*-values indicate the probability area under ROC curve values differ from 0.5. Training set and test set AUC values were statistically compared using the DeLong method. *Statistically significant

The final ensemble neural network model developed in the training set is depicted in Fig. 2A. Our analysis of relative importance suggested that neutrophil, monocyte, and basophil counts were the strongest predictors of stroke across the entire ensemble model, all exhibiting positive associations with stroke in nearly every sub-model. This was followed by platelet and lymphocyte counts, which exhibited negative associations with stroke in nearly every sub-model. Red blood cell and eosinophil counts were the weakest predictors of stroke across the entire ensemble, but did still offer high predictive value in some sub-models (Fig. 2B).

In terms of diagnostic performance, in the training set, the final ensemble model produced an AUC of 0.84 (0.95 CI: 0.78–0.89) in ROC analysis, and could discriminate between stroke patients and stroke mimics with 83.3% (0.95 CI: 69.2–91.7%) sensitivity and 70.1% (0.95 CI: 55.2–79.3%) specificity at optimal diagnostic cutoff. In the test set, the model produced an AUC of 0.78 (0.95 CI: 0.67–0.89), and could discriminate between groups with 57.5% (0.95 CI: 22.5–75.0%) sensitivity and 89.7% (0.95 CI: 58.6–96.6%) specificity. Finally, when considering the total subject pool, the final ensemble model produced an AUC of 0.82 (0.95 CI: 0.78–0.87), and could discriminate between groups with 68.1% (0.95 CI: 52.5–75.6%) sensitivity and 82.8% (0.95 CI: 68.1–89.7%) specificity. Statistical comparison of area under ROC curves revealed no statistical difference in model performance between the training and test sets (Fig. 2C).

**Fig. 3:**
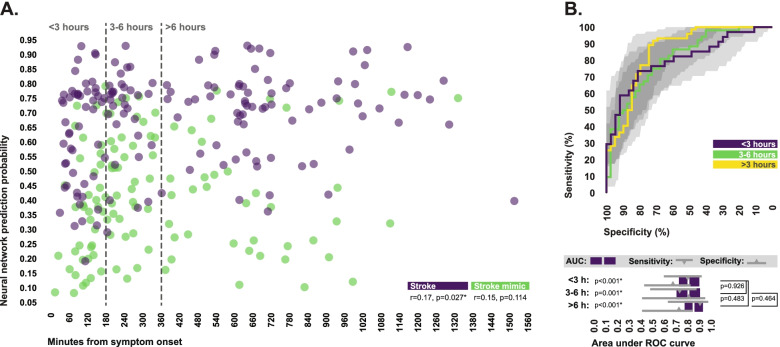
Effect of time from symptom onset on model performance. **A** Relationship between time from symptom onset to blood collection and the prediction probability of stroke produced by the final ensemble neural network model, in both stroke patients and stroke mimics, across the total study population. Strength and significance of correlations were accessed using Spearman’s rho. **B** ROC curves comparing the ability of the final ensemble neural network model to discriminate between stroke and stroke mimicking conditions in patients evaluated less than 3 h, from 3 to 6 h, or greater than 6 h from symptom onset. Sensitivity and specificity values are indicated for the diagnostic cutoff which produced the highest Youden index. Bootstrapped 95% confidence intervals associated with all diagnostic statistics are indicated. P-values indicate the probability area under ROC curve values differ from 0.5. AUC values were statistically compared across timepoints using the DeLong method. *Statistically significant

When considering the total subject pool, correlational analysis revealed a weak but statistically significant positive relationship between time from symptom onset to blood collection and the prediction probability of stroke produced by the final ensemble neural network model in stroke patients, suggesting that the model may offer slightly lower levels of sensitivity at earlier time points in the progression of pathology (Fig. 3A). Areas under ROC curves generated in subsets of patients respectively evaluated within three hours, from three to six hours, and greater than six hours from symptom onset were highly similar; however, when considering the overall shape of the ROC curves, there did appear to be a slight shift from higher specificity at the earliest timeframe towards higher sensitivity at the latest timeframe (Fig. 3B).

### Comparison of the artificial neural network model to other decision-making tools

**Fig. 4:**
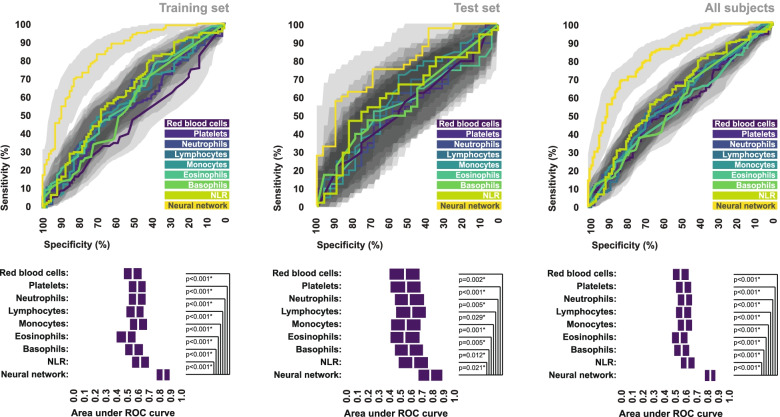
Comparison of the final neural network model to individual cell counts for stroke recognition. ROC curves depicting the ability of the final ensemble neural network model to discriminate between stroke patients and stroke mimics, in comparison to the abilities of each individual cell count, with respect to the training set, test set, and total study population. Bootstrapped 95% confidence intervals associated with area under ROC curve values are indicated. AUC values were statistically compared using the DeLong method. *Statistically significant

**Fig. 5:**
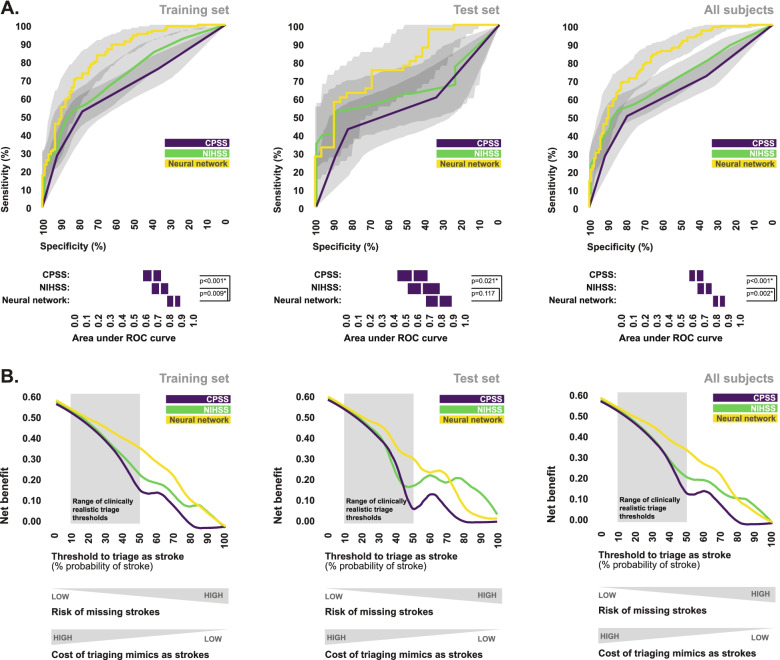
Comparison of the final neural network model to symptom-based scales for stroke recognition. **A** ROC curves depicting the ability of the final ensemble neural network model to discriminate between stroke patients and stroke mimics, in comparison to CPSS scores and NIHSS scores, with respect to the training set, test set, and total study population. Bootstrapped 95% confidence intervals associated with area under ROC curve values are indicated. AUC values were statistically compared using the DeLong method. *Statistically significant. **B** Decision curves comparing the observed net benefit of using the final ensemble neural network model as a triage tool to those of the CPSS and the NIHSS, across a range of decision thresholds

Statistical comparison of area under ROC curves revealed that the final ensemble neural network model provided a significantly greater overall level of diagnostic performance than the counts of any individual cell population, or NLR, in each of the training set, test set, and total subject pool (Fig. 4). Similarly, area under ROC curves associated with the final ensemble neural network model were significantly greater than those associated with CPSS scores across all subsets of subjects. Area under ROC curves associated with the final ensemble neural network model were also greater than those associated with NIHSS scores across all subsets of subjects, however this difference was only statistically significant in the training set and the total subject pool (Fig. 5A).

Our decision curve analysis suggested that the aforementioned differences in diagnostic performance would result in clinical benefit if the final ensemble neural network model were used as a triage decision-making tool as opposed to either the CPSS or the NIHSS, at least in our study population. It could be argued that the decision threshold to triage a patient as stroke should reasonably fall somewhere between a 10–50% probability that they are indeed having a stroke, depending on the specific triage decision (inter-hospital transfer, intra-hospital referral, etc.). In most situations, the use of a threshold over 50% would result in an unacceptable risk of triaging strokes as mimics, and the use of a threshold of less than 10% would incur unnecessary cost and resource strain associated with triaging too many mimics as strokes. Across this entire range of clinically relevant decision thresholds, the net benefit of using the final ensemble neural network model to triage stroke was greater than that of using either the CPSS or the NIHSS, in each of the training set, test set, and total subject pool (Fig. 5B).

## Discussion

The primary aim of this investigation was to determine whether machine-learning can be used to identify stroke during triage by analyzing patterns of stroke-induced changes in routinely measured circulating blood cell counts. The final ensemble neural network model generated in our analysis was able to discriminate between stroke patients and stroke mimics with relatively high levels of accuracy at emergency department admission using only information extracted from the CBC + diff. Our results suggest that machine-learning based tools could be implemented in the future to help clinicians recognize stroke in the acute phase of care via this widely available and commonly performed laboratory test.

Many of the differences in circulating cell counts which we observed between stroke patients and stroke mimics were similar to those reported in prior investigations, providing further evidence that the cellular complexion of the peripheral blood is altered in stroke. However, to our knowledge, our analysis is one of the first to make direct case–control comparisons of these cell populations between stroke patients and true stroke mimics in the acute phase of care, as opposed to previous analyses which have predominantly employed neurologically normal controls [13–15, 28–36]. It has become increasingly evident that stroke triggers a general activation of the innate arm of the peripheral immune system, and a general suppression of the adaptive arm of the peripheral immune system [11, 12]. Accordingly, numerous prior studies of both ischemic and hemorrhagic pathology have reported that stroke induces an increase in circulating numbers of neutrophils and monocytes, and a simultaneous decrease in the number of circulating lymphocytes [13, 28–31, 35–37]. Our results are highly consistent with these prior reports, as some of the most statistically significant differences we observed between stroke patients and controls were in these three cell populations. A small number of prior studies of ischemic stroke have suggested a possible drop in circulating eosinophil counts early in pathology [32, 38]; while we did observe slightly lower eosinophil counts in stroke patients versus stroke mimics, this difference was not statistically significant. To our knowledge, there have been no prior case control studies of circulating basophil counts in stroke; we observed a slight increase in basophil counts in stroke patients versus controls, but this difference was again not statistically significant. However, given that there appeared to be a relatively strong positive association between basophil count and stroke in nearly every sub-model of our neural network ensemble, the effect of stroke on basophil count may warrant further investigation.

With respect to non-immune cell populations, several prior case control studies have reported lower circulating platelet counts in patients with both ischemic and hemorrhagic stroke at hospital admission [14]. It is theorized that this phenomena is at least in part a result of stroke-related increases in platelet activation and depletion in the absence of changes in platelet production [39]. Our results were strongly supportive of these prior studies, as we too observed significantly lower platelet counts in stroke patients versus stroke mimics. There is less definitive prior evidence regarding stroke-associated changes in red blood cell counts in stroke in the acute phase of care. There is evidence that conditions which result in both abnormally high or abnormally low red blood cell counts can increase the risk of stroke [40], however there have been surprisingly few case–control analyses of red blood cell counts in stroke patients during early pathology; those that have been performed have investigated ischemic stroke specifically, and reported either similar or decreased red blood cell counts in stroke patients relative to controls [15, 33, 34]. Here, we observed slightly higher red blood cell counts in the stroke group compared to the stroke mimic group, a difference that was only statistically significant after controlling for age. Interestingly, red blood cell count was strongly positively associated with stroke in two sub-models of our neural network ensemble, and strongly negatively associated with stroke in another two; because stroke can be secondary to both conditions that result in increases or decreases in red blood cell count, it is plausible that these different sub-models are modeling different stroke etiologies, which highlights a benefit of our ensemble learning strategy.

Despite many being statistically significant, none of the aforementioned differences we observed in cell counts between stroke patients and stroke mimics were able to discriminate between groups with high levels of accuracy in isolation, nor was the NLR. However, our final ensemble neural network model was able to identify stroke with levels of diagnostic performance that exceed those of the CPSS and the NIHSS, which are two of the most widely used symptom based tools used to evaluate stroke in the emergency department. Not only does this highlight the power of machine learning-based analysis and the benefit of diagnostically considering the collective pattern of changes across the entirety of cellular populations present in the blood, but it also suggests that the strategy which we employed here could have true clinical utility. Algorithmic evaluation of the CBC + diff using machine-learning tools could be used to provide a stroke probability which could be used to help emergency department clinicians make better-informed transfer, referral, and treatment decisions in situations when stroke is expected; this could be especially useful in smaller rural hospitals where stroke expertise or advanced imaging techniques may not be immediately available [2, 3]. Perhaps more intriguing, because a CBC is ordered for a majority of emergency department patients [9], it could be used to flag individuals who may be experiencing a stroke as part of an automated stroke alert system which is integrated with the electronic medical record. Such a system could expedite care in situations where stroke is not initially being considered as a diagnosis, such as cases with atypical presentations or mild symptoms [6], and help reduce treatment lags stemming from delayed recognition in frequently mistriaged populations such as the young and minorities [41, 42].

It is pertinent to note that this is not the first work to attempt to employ machine-learning methods to develop diagnostic tools for stroke. There have been several studies published by our group and others over the past decade that have used similar methodology to develop diagnostic models; however, they have largely focused on the analysis of neuroradiological images [43, 44] or non-standard-of-care molecular biomarkers [45–48], whereas to our knowledge, this study is the first to focus solely on the analysis of circulating cell counts which are routinely assessed as part of stat testing ordered in the emergency department. The aforementioned imaging-based tools certainly have demonstrated that they can serve an important role in the diagnostic pathway, however, they only have utility if a patient is first referred to said imaging based on positive results in initial symptom-based screening. Furthermore, the aforementioned strategies developed using non-standard-of-care biomarkers in conjunction with machine-learning methods have yet to be trialed in studies using true stroke mimics, and their diagnostic performance has never been directly compared to the symptom-based tools currently used for initial triage; even if they do show strong performance in future testing, they would be expensive to implement, and would require an initial suspicion of stroke to be ordered.

While our results are exciting, further refinements and validation are needed before such strategies could be clinically implemented. While the model we developed here only considered the absolute cell counts, there is other information available as part of the CBC + diff that could also be included to potentially increase accuracy. For example, platelet and red blood cell mean volume and distribution width have been shown to be altered in stroke [14, 49], and are now generated by most hematology analyzers. Furthermore, because of the relatively small number of hemorrhagic stroke patients in our study cohort, we chose to combine both ischemic and hemorrhagic stroke patients into a single pooled stroke group; while both types of stroke appear to be associated with a similar general pattern of cellular changes to the peripheral blood, there may be subtle differences that could further differentiate between the two. For example, while neutrophil, monocyte, and lymphocyte counts are all altered in both hemorrhagic and ischemic stroke [13, 28–31, 35–37], prior studies suggest that magnitude of these alterations may be different [35, 50]. Thus, in larger future investigations where it is more feasible, these stroke subtypes could be modeled as separate groups; given that they result in slightly different blood changes, doing so may actually improve the accuracy of overall stroke recognition. Also, given the size and preliminary nature of our analysis, we chose to exclude patients with transient ischemic attack; clearly, future studies will need to include and determine how to diagnostically account for this population.

Finally, in a future larger follow-up analysis, it is possible that a different neural network implementation or other type of machine-learning algorithm may offer better performance. Here, because of relatively small sample size, we decided to use an ensemble strategy and a relatively low error threshold for neural network convergence within the composite sub-models to increase generalizability. However, in a larger sample size, such hyper-parameters may be able to be adjusted to increase accuracy without as much risk of overfitting. Furthermore, different machine-learning classifiers can be better trained and offer better performance versus others in different use scenarios depending on factors such as the degree of linearity in the relationships between predictor variables and output variables or the ratio between the number of predictor variables and number of cases. While we used artificial neural networks successfully here, follow-up investigations employing more predictors or modeling different stroke subtypes as separate groups such should also assess and empirically compare additional algorithms such as support vector machine and random forest to identify the approach which will offer the best diagnostic performance.

## Conclusions

Modeling patterns of stroke associated changes to the CBC + diff using machine-learning could yield tools with the potential to help emergency department clinicians make better informed triage decisions in situations where advanced imaging techniques or neurological expertise are not immediately available. Furthermore, the principles demonstrated in this work could provide the foundation for development of an automated stroke alert system which pulls hematology data from the electronic medical record and returns a flag to identify patients in which stroke is likely.

## Data Availability

The data that support the findings of this study are available from Valtari Bio Incorporated but restrictions apply to the availability of these data, which were used under license for the current study, and so are not publicly available. Data are however available from the corresponding author upon reasonable request and with permission of Valtari Bio Incorporated.

## Abbreviations

CBC: Complete blood count
CBC + diff: Complete blood count with differential
AUC: Area under curve
ROC: Receiver operating characteristic
CPSS: Cincinnati prehospital stroke scale
NIHSS: National Institutes of Health stroke scale
NLR: Neutrophil-to-lymphocyte ratio
